# Assessing the predictive capability of machine learning models in determining clinical outcomes for patients with cervical spondylotic myelopathy treated with laminectomy and posterior spinal fusion

**DOI:** 10.1186/s13037-024-00403-1

**Published:** 2024-06-06

**Authors:** Ehsan Alimohammadi, Elnaz Fatahi, Alireza Abdi, Seyed Reza Bagheri

**Affiliations:** 1 Department of Neurosurgery, Kermanshah University of Medical Sciences, Imam Reza hospital, Kermanshah, Iran; 2 Kermanshah University of Medical Sciences, Imam Reza hospital, Kermanshah, Iran

**Keywords:** Cervical spondylotic myelopathy, Laminectomy with posterior spinal fusion, Clinical outcome, Japanese orthopaedic association scale, Machine learning models

## Abstract

**Background:**

Cervical spondylotic myelopathy (CSM) is a prevalent degenerative condition resulting from spinal cord compression and injury. Laminectomy with posterior spinal fusion (LPSF) is a commonly employed treatment approach for CSM patients. This study aimed to assess the effectiveness of machine learning models (MLMs) in predicting clinical outcomes in CSM patients undergoing LPSF.

**Methods:**

A retrospective analysis was conducted on 329 CSM patients who underwent LPSF at our institution from Jul 2017 to Jul 2023. Neurological outcomes were evaluated using the modified Japanese Orthopaedic Association (mJOA) scale preoperatively and at the final follow-up. Patients were categorized into two groups based on clinical outcomes: the favorable group (recovery rates ≥ 52.8%) and the unfavorable group (recovery rates < 52.8%). Potential predictors for poor clinical outcomes were compared between the groups. Four MLMs—random forest (RF), logistic regression (LR), support vector machine (SVM), and k-nearest neighborhood (k-NN)—were utilized to predict clinical outcome. RF model was also employed to identify factors associated with poor clinical outcome.

**Results:**

Out of the 329 patients, 185 were male (56.2%) and 144 were female (43.4%), with an average follow-up period of 17.86 ± 1.74 months. Among them, 267 patients (81.2%) had favorable clinical outcomes, while 62 patients (18.8%) did not achieve favorable results. Analysis using binary logistic regression indicated that age, preoperative mJOA scale, and symptom duration (*p* < 0.05) were independent predictors of unfavorable clinical outcomes. All models performed satisfactorily, with RF achieving the highest accuracy of 0.922. RF also displayed superior sensitivity and specificity (sensitivity = 0.851, specificity = 0.944). The Area under the Curve (AUC) values for RF, Logistic LR, SVM, and k-NN were 0.905, 0.827, 0.851, and 0.883, respectively. The RF model identified preoperative mJOA scale, age, symptom duration, and MRI signal changes as the most significant variables associated with poor clinical outcomes in descending order.

**Conclusions:**

This study highlighted the effectiveness of machine learning models in predicting the clinical outcomes of CSM patients undergoing LPSF. These models have the potential to forecast clinical outcomes in this patient population, providing valuable prognostic insights for preoperative counseling and postoperative management.

## Background

Cervical spondylotic myelopathy (CSM) is a common degenerative neurological condition that occurs when the spinal cord is compressed due to degenerative changes or traumatic injury to the cervical spine [1, 2]. If left untreated, 20–60% of patients with CSM may experience neurological deterioration [3, 4]. Treatment options for CSM include anterior and/or posterior approaches to the spine [5, 6]. The anterior approach is typically used for patients with anterior spinal cord issues or cervical kyphosis, while the posterior approach is more commonly employed for treating multilevel stenosis and dorsal pathology [7–9].

There are two main posterior approaches for treating CSM: Laminectomy with or without posterior spinal fusion (LPSF) and laminoplasty [10, 11]. The choice between these approaches depends on individual cases and various clinical and radiological factors [7, 8]. LPSF is often recommended for patients experiencing axial neck pain, reduced cervical lordosis, and significant translational movement with flexion-extension [12, 13].

While some studies have examined predictors of clinical outcomes in CSM patients undergoing LPSF, factors such as patient age, smoking, preoperative neurological status, symptom duration, and the presence of comorbidities have been suggested to influence outcomes [12, 14, 15].

Machine learning models (MLMs) have become a valuable tool for analyzing the impact of different variables [16–18]. These models can identify patterns and relationships in data sets, making predictions based on flexible data relationships without relying on specific Eqs. [19, 20]. MLMs have increasingly been used in biostatistics and medicine to categorize and predict patient outcomes [18, 20–22]. However, there is limited research on using MLMs to predict factors influencing the clinical outcomes of CSM patients undergoing LPSF. This study seeks to evaluate the effectiveness of MLMs in predicting these factors.

## Methods

### Study population

We conducted a retrospective analysis of 329 consecutive patients diagnosed with cervical spondylotic myelopathy (CSM) based on radiological findings who underwent cervical laminectomy and fusion at our institution. The study included patients treated between Jul 2017 and Jul 2023, with approval from our institute’s ethics committee and written informed consent from all participants. Patients with a history of prior cervical surgery, cervical spine tumors, or congenital cervical deformities were excluded from the study.

### Evaluation of clinical outcomes

We assessed patients’ neurological status using the modified Japanese Orthopaedic Association (mJOA) scale before surgery and at the last post-operative follow-up. The recovery rate of myelopathy was calculated using the formula: Recovery Rate = (Postoperative JOA score - Preoperative JOA score) / (17 - Preoperative JOA score) × 100%. Previous research has indicated that a minimum clinically important difference (MCID) for JOA score recovery rate is 52.8% [14, 23]. Patients were categorized into two groups: the good group (those achieving MCID) and the poor group (those not achieving MCID). Various variables such as age, gender, BMI, smoking status, diabetes, number of laminectomy levels, MRI signal changes, symptom duration, preoperative JOA scale, Pavlov ratio, cervical curvature, and range of motion (ROM) were compared between the two groups as potential predictors for poor clinical outcomes.

### Radiological assessment

Radiological evaluations of the cervical spine, including plain radiographs, cervical computed tomography (CT) scans, and cervical MRI scans before and after surgery, were conducted. Cervical spinal curvature based on Cobb’s method and the canal-body ratio (Pavlov ratio) were measured preoperatively and postoperatively. Cervical ROM was calculated by summing the cervical angles in maximal flexion and extension on lateral radiographs. Rates of loss of cervical curvature and ROM were assessed using specific formulas [12, 14, 24].

Loss of lordosis (%) = (preoperative cervical curvature - final visit.

cervical curvature)/ (preoperative cervical curvature) ×100%.

Loss of cervical spine ROM (%) = (preoperative ROM - final visit.

ROM)/ (preoperative cervical curvature) ×100%.

Signal changes within the spinal cord were identified using T1- and T2-weighted MRI images. All radiological measurements were performed by a senior author who was blinded to the clinical outcomes.

### Statistical analysis

Data analysis was carried out using SPSS 23 software. Results were presented as mean ± standard deviation. Statistical tests including Mann-Whitney U test, independent t-test, and Pearson’s chi-square test were used to compare variables between the good and poor outcome groups. Additionally, binary logistic regression analysis was conducted to identify independent associations between prognostic factors and clinical outcomes. A significance level of *p* < 0.05 was considered statistically significant.

### Model development

In this research, we employed four machine learning models: random forest (RF), logistic regression (LR), support vector machine (SVM), and k-nearest neighbors (k-NN), to predict treatment failure in thoracolumbar burst fractures treated with SSPSF. Additionally, LR and RF models were used to analyze factors associated with treatment failure. Each model underwent training before assessment. The dataset was split into training and test sets at an 80:20 ratio. The training set was used to train the models, while the test set was used to evaluate model performance. Feature selection was based on significance in univariate analysis, with significant features from the univariate analysis serving as inputs for the machine learning techniques.

### Decision tree (DT) and random forest (RF) models

A decision tree (DT) is a tree-like structure that makes decisions based on input data, with the root node posing the initial question. Each node is connected to subsequent child nodes through branches, determining the best-split feature using a split criterion. The binary DT divides each parent node into two child nodes until all observations are classified, leading to a leaf node or outcome. Random forest (RF) is an ensemble of multiple DTs. Each tree independently predicts the outcome and votes for the most likely class. RF assigns the outcome based on the majority vote, leveraging multiple trees to make accurate predictions by capturing complex relationships. In this study, 500 DTs were utilized to construct the RF model, known for handling complex data and mitigating overfitting in classification and regression tasks [25, 26].

### Logistic regression (LR)

Logistic regression (LR) is a widely used predictive model for clinical decision-making and binary outcome classification. The LR algorithm generates a sigmoid curve to depict the relationship between inputs and outcomes, mapping inputs to probabilities (ranging from 0 to 1) that indicate the likelihood of belonging to one of two classes. By employing the logistic regression model, calculating the probability of each data point belonging to a specific outcome is straightforward. Following the determination of probabilities for each individual’s class membership, individuals are assigned to the group with the highest probability.

### Support vector machine (SVM)

Support vector machine (SVM) is a machine learning algorithm used for regression and classification tasks, finding applications in various fields such as chemometrics, bioinformatics, and biometrics. The core principle involves establishing an optimal decision boundary, represented as a line, to separate data points and minimize errors. In a two-dimensional plane, each dimension corresponds to an attribute or feature, with observations depicted as data points. The algorithm aims to create a hyperplane that effectively separates one group of points from another in a linear manner. When data is linearly separable, hyperplanes with maximum margins between points and the hyperplane are ideal for accurate predictions. In cases where data is not linearly separable, a kernel function is employed to map data to a higher-dimensional space, enabling linear separation without altering the original data. In this study, the radial basis function (RBF) kernel, known for its generalizability, was utilized [27, 28].

### K-nearest neighbors (K-NN)

The k-nearest neighbors (k-NN) algorithm is a straightforward supervised machine learning technique used for classification and regression. Its objective is to assign a data point to a class based on the nearest point in the training dataset. The predictive class is determined by the majority class among the nearest neighbors. For regression, the average value of neighboring points is used. The algorithm’s steps for classifying new data involve determining the number of nearest neighbors (k), calculating distances between new data and training data points, ranking distances, and classifying the new data based on majority votes from neighboring points.

Performance Evaluation The performance of predictive models was assessed using metrics such as accuracy, sensitivity, specificity, positive predictive value (PPV), and negative predictive value (NPV). Additionally, the area under the curve (AUC) of the receiver operating characteristic (ROC) was employed to evaluate the models’ ability to predict treatment failure [29, 30].

### Software

For statistical analysis, SPSS version 23 was used to present descriptive and inferential statistics, as well as to conduct univariate and multivariate analyses. The randomForest package was employed for fitting the RF model, the e1071 package for SVM fitting, and the caret package for calculating performance metrics. These packages are available in R4.0.3 software.

## Results

Table 1 summarizes the demographic characteristics of 329 patients who underwent posterior cervical laminectomy and fusion. The cohort comprised 185 males (56.2%) and 144 females (43.8%), with a mean age of 64.23 ± 7.21 years and an average follow-up duration of 17.86 ± 1.74 months. Among the patients, 137 individuals (41.6%) underwent ≤ 3 levels of cervical laminectomy and fusion, while 192 cases (58.4%) underwent > 3 levels (Tables 1 and 2). Table 3 presents various variables and clinical outcomes. The favorable outcome group included 267 patients with a JOA score recovery rate ≥ 52.8%, whereas the poor outcome group comprised 62 patients with a JOA score recovery rate < 52.8%. A statistically significant improvement in mJOA score was observed at the final follow-up (*P* < 0.05). Table 2 displays the mean and standard deviations of baseline and final values of radiological and clinical characteristics.

**Table 1:** Descriptive characteristics of the sample

Variable		Frequency (%)
Sex	Male	185 (56.2%)
	Female	144 (43.8%)
Clinical outcome	Poor	62 (18.8%)
	Good	267 (81.2%)
Number of laminectomy levels (n)	≤ 3	137 (41.6%)
	> 3	192 (58.4%)
Signal changes in MRI	No change in signal intensity	77 (23.4%)
	Signal change on T1- + T2-weighted images	146(44.4%)
	Signal change on T2-weighted images	106 (32.2%)
Smoking	Yes	95 (28.9%)
	No	231 (71.1%)
Diabetes Mellitus	Yes	100 (30.4%)
	No	229 (69.6%)

**Table 2:** Mean and standard deviation of quantitative variables

Variable	Mean	Standard Deviation
Age	64.23	7.21
Follow Up(months)	17.86	1.74
Duration of symptoms(months)	10.21	2.23
Body Mass Index	24.01	1.59
Preoperative JOA scale	12.21	1.42
Final JOA scale	15.18	1.54
Preoperative Pavlov ratio	0.71	0.022
Final Pavlov ratio	0.98	0.02
Preoperative Curvature (°)	13.93	1.24
Final Curvature (°)	11.02	1.41
Preoperative Cervical spine ROM(°)	34.31	5.18
Final Cervical spine ROM(°)	15.33	4.51

**Table 3:** Relationship between qualitative variables and clinical outcomes

Variable		Clinical outcomes		Statistical Analysis
		Favorable outcome N (%)	Unfavorable outcome N (%)	
Sex	Male	149 (80.5)	36 (19.5)	*P* = 0.325
	Female	118 (81.9)	26 (18.1)	
Number of laminectomy levels (n)	≤ 3	115 (83.9)	22 (16.1)	*P* = 0.237
	> 3	152 (79.2)	40(20.8)	
Signal changes in MRI	No change in signal intensity	74 (96.1)	3 (3.9)	* ***P*** ** < 0.001**
	Signal change on T1- + T2-weighted images	105 (71.9)	41 (28.1)	
	Signal change on T2-weighted images	88 (83.00)	18(17.00)	
Smoking	Yes	77 (81.3)	18(18.8)	*P* = 0.401
	No	190(81.2)	44 (18.8)	
Diabetes Mellitus	Yes	75 (75.00)	25 (25.00)	*P* = 0.163
	No	192 (83.8)	37 (16.2)	

### Predictors of poor clinical outcome based on univariate analysis

In our investigation, age, preoperative mJOA scale, symptom duration, and MRI signal changes were identified as predictors of poor clinical outcome in the univariate analysis (*p* < 0.05) (Tables 3 and 4). No association was found between clinical outcome and gender, number of laminectomy levels, smoking status, diabetes mellitus, BMI, preoperative Pavlov ratio, preoperative cervical curvature, and preoperative cervical spine range of motion (Tables 3 and 4).

**Table 4:** Relationship between clinical outcomes and quantitative variables

Variable	Clinical outcomes			Statistical Analysis
	Good outcome *N* (%)	Poor outcome *N* (%)		
Age (year)	60.73 ± 6.22		68.24 ± 5.61	* ***P*** ** < 0.001**
Follow Up (months)	17.49 ± 1.43		18.32 ± 1.42	*P* = 0.421
Duration of symptoms(months)	8.22 ± 1.64		15.11 ± 1.57	* ***P*** ** = 0.023**
Body Mass Index	24.27 ± 1.40		23.73 ± 1.72	*P* = 0.522
Preoperative mJOA scale	12.66 ± 1.34		10.39 ± 1.35	* ***P*** ** = 0.002**
Final mJOA scale	15.32 ± 1.23		12.22 ± 1.19	* ***P*** ** = 0.009**
Preoperative Pavlov ratio	0.74 ± 0.06		0.76 ± 0.21	*P* = 0.122
Final Pavlov ratio	0.97 ± 0.7		0.96 ± 0.23	*P* = 0.227
Preoperative Curvature (°)	14.55 ± 1.20		13.78 ± 1.13	*P* = 0.343
Final Curvature (°)	11.29 ± 1.42		10.44 ± 1.47	*P* = 0.288
Preoperative Cervical spine ROM(°)	36.15 ± 1.73		33.55 ± 1.52	*P* = 0.552
Final Cervical spine ROM(°)	15.84 ± 1.63		14.67 ± 1.42	*P* = 0.330

### Predictors of poor clinical outcome based on multivariate analysis

Binary logistic regression analysis revealed that age (odds ratio [OR] 2.08; 95% confidence interval [95% CI] 1.47–2.54; *P* = 0.013), preoperative mJOA scale (OR 3.52; 95% CI 2.83–4.56; *P* < 0.001), and symptom duration (OR 1.37; 95% CI 1.01–2.01; *P* = 0.031) were independent predictors of poor clinical outcome (Table 5).

**Table 5:** Binary Logistic Regression Analysis

variables	Odds ratio	95% CI	*P* value
Age	2.08	1.47–2.54	* ***P*** ** = 0.013**
Signal changes in MRI	1.24	0.83–1.91	*P* = 0.421
Duration of symptoms	1.37	1.01–2.01	* ***P*** ** = 0.031**
Preoperative mJOA scale	3.52	2.83–4.56	* ***P*** ** < 0.001**

Each machine learning model utilized feature selection to assess the independent significance of risk factors. According to the mean Gini index, the RF model identified preoperative mJOA scale, age, symptom duration, and MRI signal changes as the most crucial variables in descending order. The study evaluated the predictive accuracy of LR, RF, SVM, and k-NN models for poor clinical outcomes. RF demonstrated the highest accuracy of 0.922, followed by SVM at 0.901, k-NN at 0.887, and LR at 0.876, respectively. RF also showed superior sensitivity and specificity compared to the other models (sensitivity = 0.851, specificity = 0.944). LR, SVM, and k-NN predicted poor clinical outcomes with negative predictive values (NPVs) of 0.849, 0.803, and 0.794, respectively. The AUC values for RF, LR, SVM, and k-NN were 0.905, 0.827, 0.851, and 0.883, respectively (Table 6).

**Table 6:** Evaluation criteria for comparison performance of machine learning models (LR, RF, SVM and k-NN)

Evaluation criteria	Model			
variables	RF	LR	SVM	K-NN
Accuracy	0.922	0.876	0.901	0.887
Sensitivity	0.851	0.675	0.733	0.804
Specificity	0.944	0.918	0.909	0.923
Positive predictive value	0.722	0.783	0.701	0.688
Negative predictive value	0.884	0.849	0.803	0.794
AUC	0.905	0.827	0.851	0.883

RF: Random forest; LR: Logistic regression; SVM: Support vector machine; k-NN: k- nearest neighbor; AUC: area under the curve of mean receiver operating characteristics

## Discussion

Our results showed that age, preoperative mJOA scale, and duration of symptoms were predictors of poor clinical outcome. There are several studies that evaluated the relationship between preoperative severity of myelopathy/duration of myelopathy symptoms with the clinical outcome of patients with CSM [2, 14]. The majority of evidence has revealed a significant predictive value for the severity of preoperative myelopathy and duration of myelopathy symptoms in the clinical outcomes of these patients [8, 15]. However, some studies reported no significant relationship between the modified Japanese Orthopaedic Association (mJOA) or JOA recovery rate after operation and the severity of baseline myelopathy symptoms [31]. In a retrospective study, Gao et al. assessed the clinical outcome of 145 consecutive patients undergoing surgery for CSM, with a mean followup of 5 years. Their results showed that subjects with a preoperative JOA of ≤ 9 were 4.84 times more likely to exhibit a “fair” outcome (< 50% recovery rate) in comparison with those with a JOA > 9 [32]. Furthermore, Pumberger et al. showed that cases with less severe myelopathy on the Nurick grading system (≤ 3) were more likely to achieve a grade of 0, 1, or 2 after surgery in comparison with those with a baseline grade of ≥ 4. Moreover, they reported that cases with symptom duration of less than 1 year were 4.8 times more likely to improve and 14 times more likely to return to a Nurick grade of 0 after operation when compared to the subjects with symptom duration of more than 1 year [33]. There is a controversy on the impact of the age on clinical outcome of patients with CSM [5]. Our results showed that advanced age was associated with poor outcome. Although the majority of evidence demonstrated a significant predictive value for age in patients with CSM, some studies revealed no association between age and outcomes in terms of mJOA, Nurick, and SF-36 scores [5, 34]. Based on the analysis of the Cervical Spondylotic Myelopathy (CSM)North America and CSMInternational datasets, Tetreault et al. reported that patients with advanced age were less likely to achieve an mJOA score ≥ 16 at 12 months or achieve a MCID after operation for CSM [10]. One hypothesis that could explain this finding is that people who are younger and less severely affected have minor neuropathologic changes in the spinal cord [10, 14].

The objective of this study was to employ machine learning models to predict factors associated with poor clinical outcomes of patients with CSM who underwent LPSF. The results presented in Table 6 indicate that all machine learning models performed well, with Random Forest (RF) demonstrating superior performance across all criteria in predicting treatment failure with the least amount of error. When comparing the classification ability of the evaluated models, RF outperformed the others. RF is an ensemble learning method that combines multiple decision trees to make predictions. Several characteristics contribute to its superior performance [25, 35]. Firstly, the ensemble approach helps mitigate overfitting and enhances the model’s generalization ability by combining predictions from different subsets of the data. Secondly, RF provides a measure of variable importance, identifying the relative contribution of each input variable in making predictions. This feature aids in identifying influential factors associated with treatment failure. Additionally, RF is capable of capturing complex nonlinear relationships, handling outliers and missing data, and does not assume a specific data distribution, making it suitable for analyzing complex datasets without strict assumptions [35, 36]. The study found that all models demonstrated acceptable performance in terms of the area under the curve (AUC), yielding reliable predictions without sacrificing sensitivity and specificity. However, it was noted that the performance of the predicting models is dependent on the training dataset, and partiality in training can introduce bias. The study used 80% of the data for training and 20% for testing, but acknowledged that a larger dataset would help reduce bias. Missing data was identified as an important limitation, but in this study, there was no missing data due to meticulous physical exams and clinical evaluations.

### Limitations

Limitations of the study should be considered when interpreting the findings and their clinical implications. The retrospective design and reliance on existing medical records may lead to incomplete or missing data, potentially limiting the ability to account for all relevant variables and confounders. Additionally, the study was conducted at a single center, potentially limiting the generalizability of the findings. Although the study included 329 subjects, a larger sample size would enhance statistical power and generalizability. While the machine learning models demonstrated satisfactory predictive performance, their interpretability may be limited. Understanding the specific factors driving the predictions of these models can be challenging, potentially affecting their clinical utility and decision-making process. Prospective studies with standardized data collection protocols would provide more robust and comprehensive results.

## Conclusions

This study demonstrated the efficacy of machine learning models in predicting the clinical outcomes of patients with CSM who underwent LPSF. The findings underscore the capacity of these models to anticipate clinical results in this particular patient cohort, offering invaluable prognostic information for guiding preoperative discussions and postoperative care.

## Data Availability

The datasets generated and/or analyzed during the current study are not publicly available due to them containing information that could compromise research participant privacy/consent but are available from the corresponding author on reasonable request.
